# MR-KPA: medication recommendation by combining knowledge-enhanced pre-training with a deep adversarial network

**DOI:** 10.1186/s12859-022-05102-1

**Published:** 2022-12-19

**Authors:** Shaofu Lin, Mengzhen Wang, Chengyu Shi, Zhe Xu, Lihong Chen, Qingcai Gao, Jianhui Chen

**Affiliations:** 1 Faculty of Information Technology, Beijing University of Technology, Beijing, China; 2 Beijing International Collaboration Base on Brain Informatics and Wisdom Services, Beijing University of Technology, Beijing, China; 3 Beijing Key Laboratory of MRI and Brain Informatics, Beijing University of Technology, Beijing, China; 4 Engineering Research Center of Intelligent Perception and Autonomous Control, Ministry of Education, Beijing, China; 5 Engineering Research Center of Digital Community, Ministry of Education, Beijing, China

**Keywords:** Medication recommendation, Electronic medical record, Graph attention network, Pre-training model, Adversarial training

## Abstract

**Background:**

Medication recommendation based on electronic medical record (EMR) is a research hot spot in smart healthcare. For developing computational medication recommendation methods based on EMR, an important challenge is the lack of a large number of longitudinal EMR data with time correlation. Faced with this challenge, this paper proposes a new EMR-based medication recommendation model called MR-KPA, which combines knowledge-enhanced pre-training with the deep adversarial network to improve medication recommendation from both feature representation and the fine-tuning process. Firstly, a knowledge-enhanced pre-training visit model is proposed to realize domain knowledge-based external feature fusion and pre-training-based internal feature mining for improving the feature representation. Secondly, a medication recommendation model based on the deep adversarial network is developed to optimize the fine-tuning process of pre-training visit model and alleviate over-fitting of model caused by the task gap between pre-training and recommendation.

**Result:**

The experimental results on EMRs from medical and health institutions in Hainan Province, China show that the proposed MR-KPA model can effectively improve the accuracy of medication recommendation on small-scale longitudinal EMR data compared with existing representative methods.

**Conclusion:**

The advantages of the proposed MR-KPA are mainly attributed to knowledge enhancement based on ontology embedding, the pre-training visit model and adversarial training. Each of these three optimizations is very effective for improving the capability of medication recommendation on small-scale longitudinal EMR data, and the pre-training visit model has the most significant improvement effect. These three optimizations are also complementary, and their integration makes the proposed MR-KPA model achieve the best recommendation effect.

## Introduction

Electronic medical records (EMRs) represent a patient’s historical visit sequence, where each sequence contains a series of clinical events (diagnosis, procedure, medication, etc.) for a single admission. More and more attention has been paid to EMR-based auxiliary diagnosis and treatment, such as clinical knowledge question answering [1, 2], health risk warning [3–6], auxiliary diagnostic [7, 8] and electronic prescription recommendation [9, 10]. Medication recommendation is an important research direction in EMR-based applications. Given a patient’s current clinical events and history of visits, the goal of the medication recommendation task is to provide a personalized combination of medications appropriate to his or her health status. It is a crucial data mining task for an intelligent healthcare system [11] and many important recommendation models have been developed [12–16].

Existing EMR-based medication recommendation methods are mainly data-driven and adopt machine learning methods, especially deep networks, to model on various clinical event sequences. In order to improve the accuracy of recommendation, related studies mainly adopted longitudinal sequential recommendation methods which integrated patient’s current health conditions and historical visit information to effectively leverage the temporal dependencies among clinical events for medication recommendation [13, 17]. Recent studies focused on developing novel and complex neural networks to capture deep-level data features, including complete structure information [11], drug-drug interactions [12], multiple-level importance [18], relationships between historical and current diagnoses [19], irregular time-series dependencies [20], for improving recommendation capabilities.

However, some diseases may require multiple follow-up visits while others do not. Patients may also visit different hospitals each time resulting in incomplete multiple-visit records. So patients’ longitudinal EMR data with multiple visits are relatively few. For example, in the experiment we collected a total data of 151,908 EMRs but only 10,448 EMRs were involved with multiple visits. The longitudinal data only account for 6.9 $\%$ of the total data. They are often discontinuous and can lead to information bias in research [21]. The lack of longitudinal data has become an important challenge for EMR-based medication recommendation.

Few-shot learning, which use small sample data for effective model training, is a current research hot spot. Related methods are divided into three categories usually, including fine-tuning, data enhancement and migration [22]. Data enhancement methods [23] usually need high-quality domain knowledge bases and are easy to introduce noise. Migration methods [24] need a group of labeled data in the similar fields for transfer learning. Hence, fine-tuning methods [25], especially pre-training [26], have become the main means for few-shot learning of EMR-based models. At present, EMRs or EHRs pre-training is attracting attentions [27–29]. However, existing EMRs pre-training methods need a large number of unlabeled data, which have the same source as labeled data, and neglect the optimization of the fine-tuning process. They also only focus disease prediction tasks whose number of classifications is far lower than medication recommendation tasks. Therefore, these existing EMRs pre-training methods cannot be used directly to solve the problem of lacking longitudinal data in EMR-based medication recommendation.

Based on the above observations and our previous study [30], this paper proposes a MR-KPA model which combines knowledge-enhanced pre-training with a deep adversarial network to realize medication recommendation based on small-scale longitudinal EMR data. The main contributions can be summarized as follows:

- Firstly, a knowledge-enhanced pre-training visit model is proposed to realize domain knowledge-based external feature fusion and pre-training-based internal feature mining for improving medication recommendation on small-scale longitudinal EMR data. Different from existing EMRs pre-training methods, this visit model uses a large number of single-visit EMR data for pre-training, in order to avoid splitting longitudinal EMR data that is already insufficient.
- Secondly, a medication recommendation model based on the deep adversarial network is developed to apply EMRs pre-training to medication recommendation for the first time. By introducing adversarial training, the fine-tuning process of pre-training visit model can be optimized to alleviate over-fitting of model caused by the task gap between pre-training and recommendation.
- Finally, a group of experiments have been performed based on real EMR data from medical and health institutions in Hainan Province, China. Experimental results show that the proposed method can effectively improve the accuracy of medication recommendation based on small-scale longitudinal EMR data.

The rest of this paper is organized as follows. “Related work” section introduces related work. “Medical codes and data sets” section describes medical codes and data sets. “Method” section introduces the proposed MR-KPA model. In “Experiment” and “Discussion” sections, the predictive performance of this model is compared and analyzed with baselines and variants. Finally, “Conclusion” section gives the conclusions and future work.

## Related work

Leveraging recommendation algorithms [31, 32]to recommend rational and effective medications in time for patients, as a paramount recommendation task in the health domain, has been widely researched [11]. Existing methods are mainly data-driven and depended on large amounts of EMR data.

Early approaches often adopted instance-based methods, which only focused on current health conditions and failed to make full use of historical information. Syed-Abdul et al. [33] proposed a smart medication recommendation model for the electronic prescription. In order to reduce the probability of illegal prescription, this smart model adopted the association rule mining technology to find the relationship between two labels for reducing the probability of illegal prescription. Zhang et al. [34] proposed the LEAP model to predict combination of medicines by giving patient’s diagnoses. This LEAP model is a variant of sequence-to-sequence model based on content-attention mechanism and, focuses on modeling mappings between instances and tag dependencies.

Obviously, patients’ historical EMR data can help to do medication recommendation. At present, studies on EMR-based medication recommendations mainly adopt longitudinal sequential recommendation methods which recommend medications based on both current health conditions and historical information [12, 17]. Choi et al. used a two-level neural attention model to detect influential past visits and significant clinical variables within those visits for improved medication recommendation [17]. An et al. proposed a relational perception LSTM (R-LSTM) to deal with the relationship between diseases and medications in longitudinal medical records, which can better integrated historical information into medication level patient representation [13]. Wang et al. proposed the adversarially regularized model for medication recommendation (ARMR), which built a key-value memory network based on information from historical admissions and carried out multi-hop reading on the memory network to recommend medications [12]. An et al. proposed a multilevel selective and interactive network (MeSIN) which fully leveraged the inherent multilevel structure of EHR data to learn a comprehensive patient representation for reasonable medication recommendation [11].

Table 1 gives a comparison of the above EMRs-based medication recommendation methods. As shown in this table, existing studies on longitudinal sequential medication recommendation mainly focused on developing different deep neural networks to capture deep-level features in EMR data. Such approaches depended on massive longitudinal EMR data. Therefore, the lack of longitudinal EMR data has become an important challenge of EMR-based medication recommendation. At present, medication recommendation based on relatively small-scale longitudinal EMR data is not given enough attention. The studies on few-shot learning of EMRs-based models mainly focused on pre-training of EMRs or EHRs data in disease prediction tasks. Various EMRs or EHRs pre-training tasks are designed to learn feature expression from large-scale unlabeled data through a self-supervised learning method [26]. For examples, Rasmy et al. [27] proposed Med-BERT, which adapted the BERT framework originally developed for the text domain to the structured EHR domain. Fine-tuning experiments on two clinical databases showed that Med-BERT can benefit disease prediction studies with small local training datasets, reduce data collection expenses, and accelerate the pace of artificial intelligence aided healthcare. Ren et al. proposed [28] a novel model RAPT, which stands for representation by Pre-training time-aware Transformer, and devise three pre-training tasks to handle data insufficiency, data incompleteness and short sequence problems. Extensive experimental results for four downstream tasks have shown the effectiveness of the proposed approach. Meng et al. [29] presented a temporal deep learning model to perform bidirectional representation learning on EHR sequences with a transformer architecture and the pre-training task of masked language modeling to predict future diagnosis of depression. However, these EMRs pre-training methods cannot be used directly to solve the problem of lacking longitudinal EMR data in EMR-based medication recommendation:

- In data, existing EMRs pre-training methods relied on a large number of unlabeled data, which have the same source as labeled data. The existing researches above usually split experimental data and use most of them for pre-training. This method of obtaining pre-training data is not applicable to longitudinal EMR data that is lacking in itself.
- In the downstream task, existing EMRs pre-training methods mainly aiming at disease prediction, which is usually a binary classification problem. On the contrary, there are often hundreds of classifications in medication recommendation. Therefore, the application of EMRs pre-training in medication recommendation should be studied separately.
- In the fine-tuning process, existing EMRs pre-training methods focused on pre-training tasks and neglected the optimization of the fine-tuning process. However, the gap between pre-training and downstream tasks can bring the catastrophic forgetting problem [35, 36]. With the increase of the number of fine-tuning iterations, the downstream tasks increasingly focuses on labelled data and leads to over-fitting of model. Therefore, it is necessary to improve the downstream models for optimizing the fine-tuning process of pre-training model.

**Table 1:** A Comparison of EMRs-based medication recommendation methods

Method/reference	Classification	Shallow/deep learning	Strategy	Data size
Smart Model [33]	Instance-based	Shallow learning	MPR$^{a}$ +CR$^{b}$	103,480,000
LEAP [34]	Instance-based	Deep learning	Recurrent Decoder	50206(Mimic-3), 2415414 (Sutter)
Retain [17]	Longitudinal sequential	Deep learning	RNN$^{c}$	14,366,030
RAHM [13]	Longitudinal sequential	Deep learning	R-LSTM$^{d}$	/
ARMR [12]	Longitudinal sequential	Deep learning	MedRec$^{e}$ +GAN	Over 40,000(Mimic-3)
MeSIN [11]	Longitudinal sequential	Deep learning	InLSTM$^{f}$ +ASM$^{g}$ +GSFM$^{h}$	11,809(Mimic-3)

$^{a}$Mean Prescription Rank

$$^{b}$$Coverage Rate

$^{c}$Recurrent Neural Networks

$^{d}$Relation-aware LSTM

$^{e}$The module contains the encoder and memory network

$^{f}$Interactive Long-short Term Memory Network

$^{g}$ Attentional Selective Module

$^{h}$A global selective fusion module

In addition, the fusion of knowledge and big data is a recent research hotspot. Integrating formal domain knowledge, such as term ontology [37, 38], knowledge graph (KG) [39, 40] and so on into deep neural networks has become an important approach to improve feature expression in various applications of deep learning, such as finance [41] and medicine [42]. For EMR-based medication recommendation, fusing domain knowledge to improve feature expression of EMR has also received attention. For an example, Choi et al. represented the medical concept as a combination of its ancestors in the medical ontology using an attention mechanism for enriching the input of EMR-based medication recommendation [17]. However, their studies still only depended on longitudinal EMR data. Though medical concepts enriched feature expression of EMR, model training still needed a large number of EMR data. The training datasets in Choi et al.’s study included three data sets, Sutter PAMF, Mimic-III and Sutter heart Failure (HF) cohort, in which the numbers of visit records were 13920759, 19911 and 572551 respectively.

In order to improve robustness and interpretability of the models, knowledge enhanced pre-training models (KEPTMs) are attracting attention. Yang et al. [43] categorized existing KEPTMs into three groups: entity enhanced pre-trained models [44, 45], triplet enhanced pre-trained models [46, 47] and other knowledge enhanced pre-trained models [48, 49]. However, all of these KEPTMs were oriented to text corpora. Though Shang et al. [16] proposed G-Bert which modified Bert pre-training tasks to realize knowledge-enhanced pre-training on large-scale single-visit EMR data, G-Bert only considered two types of medical codes and pre-training tasks only focused on themselves and their relations of medical codes. Other important information, especially symptoms, and its prediction ability for medication recommendation were not considered in pre-training tasks. Moreover, their researches also neglected the gap between pre-training and downstream tasks, which is particularly serious when labelled data are obviously smaller than unlabeled pre-training data. As stated above, longitudinal EMR data only account for 6.9$\%$ of the total data and the remaining 93.1$\%$ were single-visit data, which was indeed the case. Therefore, it is necessary to improve the recommendation model for optimizing the fine-tuning process of the single-visit pre-training model.

Based on the above analysis, we propose the MR-KPA model which combines knowledge-enhanced pre-training with a deep adversarial network to improve medication recommendation from both feature expression and recommendation model structure, for realizing medication recommendation based on small-scale longitudinal EMR data. The details are introduced in the following sections.

## Medical codes and data sets

### Medical codes

**Fig. 1:**
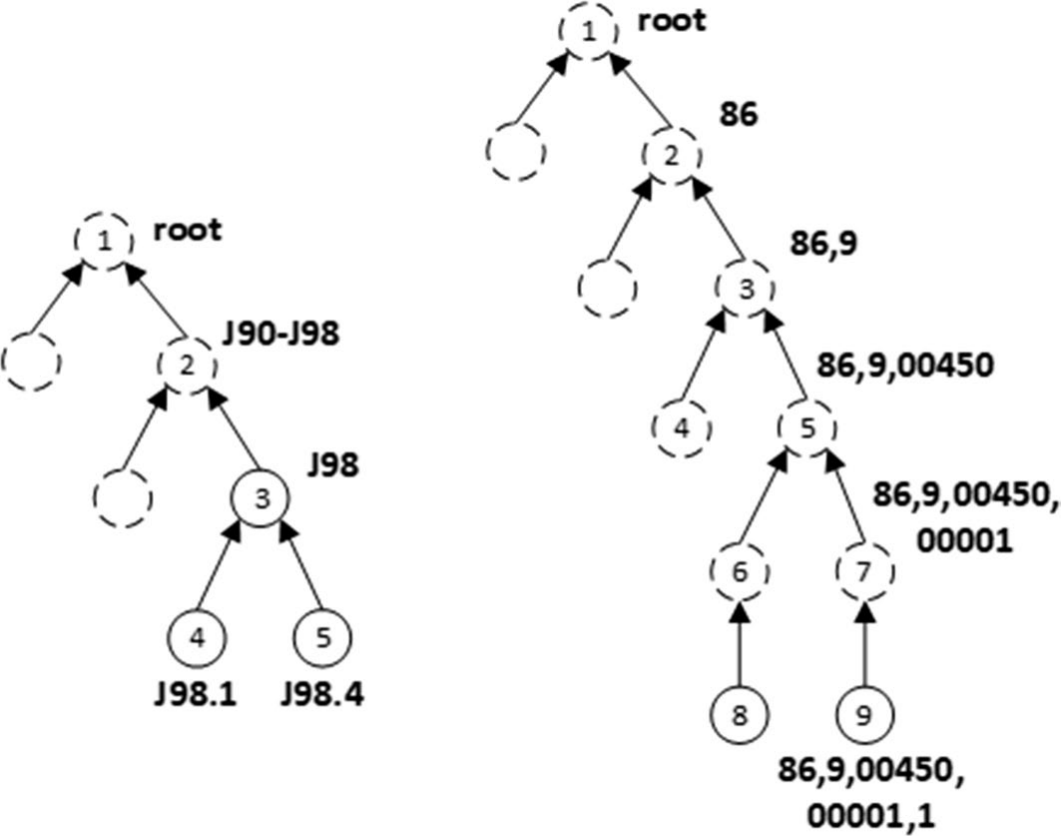
Tree structures of ICD-10 and NDC

Medical codes are usually categorized according to a tree-structured classification system for diagnosis and drug. Figure 1 gives tree structures of ICD-10 ontology and NDC ontology. All codes are the lowest leaf nodes.

The left of Fig. 1 is an example of ICD-10 J98.4 which is the ICD-10 code of “other lung diseases” and its sibling node J98.1 is the ICD-10 code of “Pulmonary collapse”. They have a common parent node J98. This means that both these two kinds of diseases belong to “other respiratory diseases” whose ICD-10 is J98.

The right of Fig. 1 is an example of NDC(Chinese National Drug Code). 86900450000011(86,9,00450,00001,1) is the NDC code of “Ceftazidime for Injection”. The codes in line with Chinese national drug coding standards have 14 digits. The first 2 digits “86” are the drug country code and the third digit “9” represents the drug category code. The fourth to eighth “00450” represents the enterprise identifier and the ninth to thirteenth “00001” represents the product identifier. The last digit “1” represents different drugs.

### Data sets

In this study, the real EMRs are from medical and health institutions in Hainan Province, China. Six important fields, PATIENT ID, INPATIENT FORM NO, OUTPATIENT DIAG CODE, DRUG STANDARD CODE and CHIEF COMPLAINTS are extracted from the IN SUMMARY DISCHARGE DIAG table, IN SUMMARY DRUG DETAIL table and ADMISSION INFORMATION RECORD table in the electronic medical record system. PATIENT ID records the patient’s unique ID and INPATIENT FORM NO records the unique ID of one visit to hospital. OUTPATIENT DIAG CODE records the ICD-10 codes of diagnosis, DRUG STANDARD CODE records NDC codes of drug and CHIEF COMPLAINTS records the patient’s current symptoms. This study uses word segmentation to divide symptom description sentence into words, and then remove pause words during word segmentation to create the symptom set of each EMR. Table 2 gives the data statistics. The single-visit records were used for training the pre-training model and the multiple-visit records were used for training and testing the prediction model. Compared with those data sizes in Table 1, our data set is very small.

**Table 2:** Statistics of the data set

Statistical field	Single-visit	Multiple-visit
Total number of records	141,460	3155
Number of patients	141,460	1390
Number of diagnosis codes	1946	754
Number of drug codes	12,993	1467
Number of symptoms	6523	2016
Avg number of diagnosis codes	2.233	1.400
Avg number of drug codes	16.77	3.943
Avg number of symptoms	1.000	0.774
Avg number of visit	1.0	2.270
Max number of diagnosis codes	26	30
Max number of drug codes	345	69
Max number of symptoms	2	7
Max number of visit	1	8

## Method

### An overview

A longitudinal sequential medication recommendation task can be defined as follows:

$\textit{Definition 1: Longitudinal EMR Data.}$ In EMR data, each patient’s records can be represented as a sequence of multivariate observations: $S^{n}=\left\{ P_1^{(n)},P_2^{(n)},\cdots P_{T^{(n)}}^{(n)} \right\}$ where n represents the n-th patient and $T^{(n)}$ is the number of visits of the n-th patient. The EMR record of the t-th visit is described as $P_t^{(n)}=\left\{ d_t^{(n)},m_t^{(n)},s_t^{(n)}\right\}$ where $d_t^{(n)}$ is a collection of diagnostic codes for ICD-10, $m_t^{(n)}$ is a collection of drug codes for National Drug Codes (NDC), $s_t^{(n)}$ is the collection of self-reported symptoms named as SYM, such as “fever”.

$\textit{Definition 2: Longitudinal Sequential Medication Recommendation Task.}$ Given the n-th patient’s history EMR records $S_{1:t-1}^{(n)}=\left\{ P_1^{(n)},P_2^{(n)},\cdots P_{t-1}^{(n)} \right\}$, diagnostic codes $d_t^{(n)}$, drug codes $m_t^{(n)}$ and symptoms $s_t^{(n)}$ at the t-th visit, we want to recommend the drugs at the t-th visit by generating multi-label output $\hat{y_t}\in \left\{ 0,1\right\} ^{ML}$which ML represents the number of drug codes. That is to say, the output of the medication recommendation is a list of appropriate drugs. And the recommendation problem is transformed to a multi-label classification problem.

This study proposed a MR-KPA model to realize this task based on small-scale data. On the one hand, the proposed model adopts a knowledge-enhanced pre-training. A large number of single-visit EMR data is used as the pre-training data for avoiding segment limited longitudinal EMR data. The classification knowledge of diagnostic and drug codes was encoded as external domain features and then fused into EMR embeddings. On the other hand, this model integrated adversarial training into multi-layer perceptron (MLP) to avoid the over-fitting of model during the fine-tuning process.

**Fig. 2:**
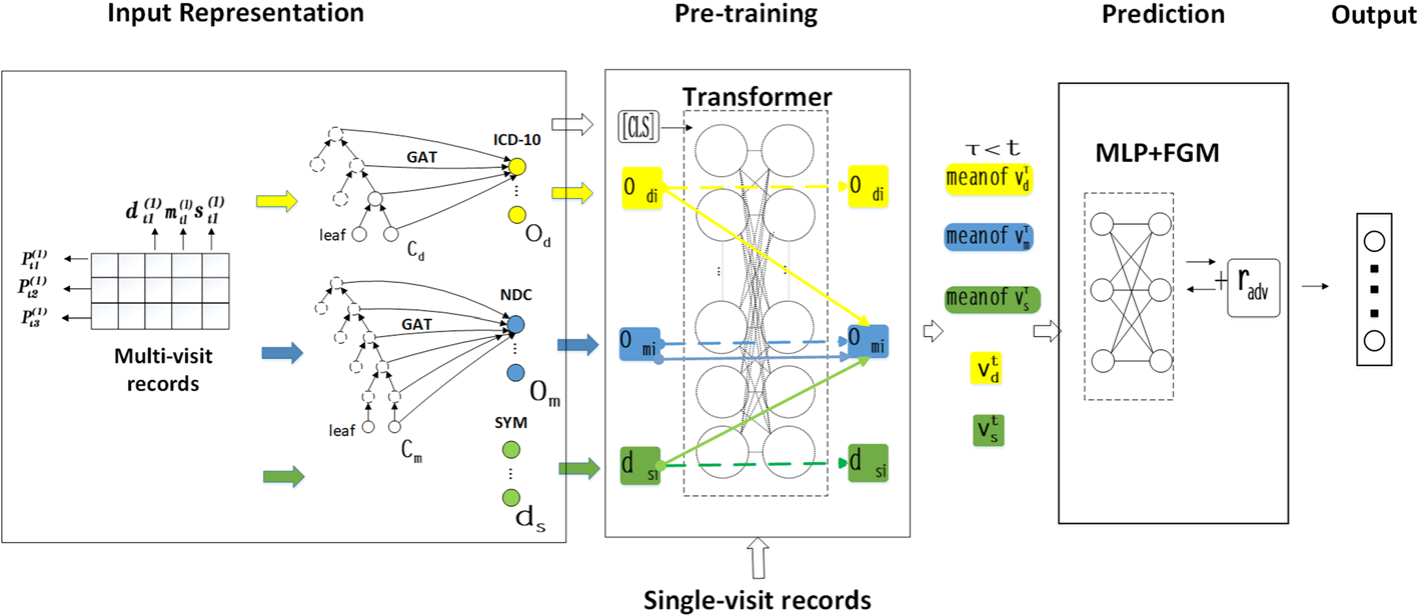
The whole framework of MR-KPA. It includes three modules: the input representation, pre-training and prediction

The whole framework of MR-KPA is described in Fig. 2. It includes three modules: input representation, pre-training and prediction. The input representation module transforms each EMR record into the diagnosis code embedding, the drug code embedding and the symptom embedding. Based on these three types of embeddings, the pre-training module creates a pre-training visit model by performing two types of pre-training tasks. Finally, the prediction module fine-tunes the pre-training visit model and obtains the predicted drug code based on patient’s multiple-visit records. The details will be described in the following subsections.

### Input representation

**Fig. 3:**
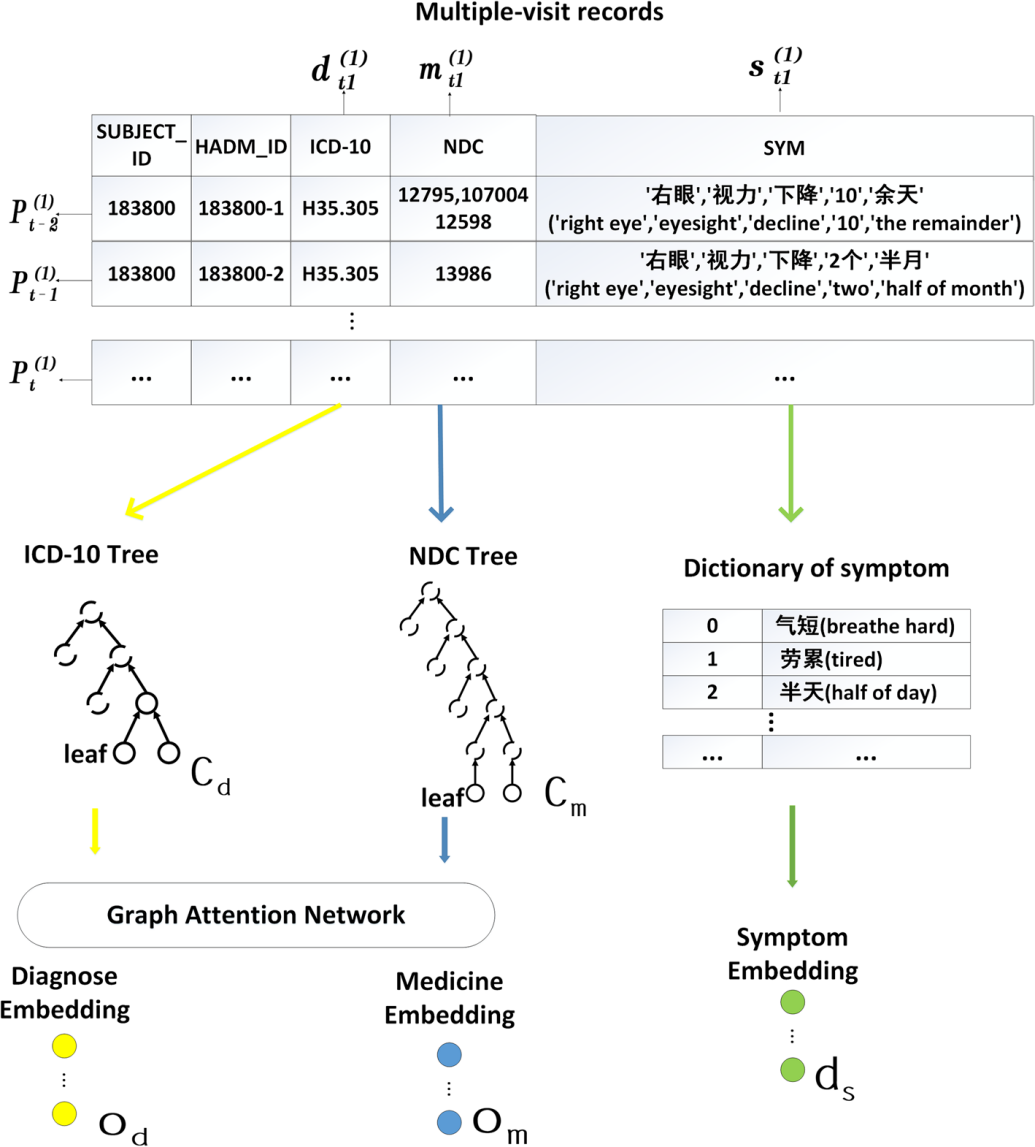
The framework of Input Representation. Both diagnose embedding and medicine embedding adopt ontology embeddings based on code trees. Symptom embedding adopts the dictionary embeddings

The input representation module transforms each EMR into a group of multi-dimensional embeddings as the input of the subsequent module. As shown in Fig. 3, multiple-visit records are inputted into this module. Each record includes columns SUBJECT ID, HADM ID, ICD-10, NDC, and SYM, which represent the patient ID, hospital ID, diagnostic code, drug code, and symptom participle respectively. They are transformed into two ontology embeddings and one dictionary embedding.

For the EMR of n-th patient at t-th visit $P_t^{(n)}=\left\{ d_t^{(n)},m_t^{(n)},s_t^{(n)}\right\}$, its input embedding can be obtained as follows.

$\textit{Ontology embedding.}$ Ontology embedding is adopted to realize domain knowledge-based external feature fusion. Two types of code ontology embeddings are constructed from ICD-10 ontology $O_{d}$ and NDC ontology $O_m$. Because medical codes in raw EMR data are leaf nodes in code ontology trees, code ontology embedding can be obtained by using graph attention network (GAT) [8, 10, 12, 13]. It can encode the classification knowledge in diagnostic and drug code trees as external domain features. For each medical code $c_*\in d_t^{(n)} \cup m_t^{(n)}$ is the embedding dimension, and then the procedure is performed to obtain its ontology embedding as follows:

1 $$\begin{aligned} o_{c_*}=g(c_*,pa(c_* ),H_e)=\parallel _{k=1}^{k}\sigma \left( \sum _{j\in N_{c_*}}a_{c_*,j}^k W^k h_j\right) \end{aligned}$$

where $*\in \left\{ d,m\right\}$, $N_{c_*}=\left\{ \left\{ c_*\right\} \cup \left\{ pa(c_* )\right\} \right\}$ are the parent nodes of $c_*$ and itself, $\parallel$ represents concatenation which enables the multi-head attention mechanism, $\sigma$ is a nonlinear activation function, $W^k \in {\mathbb {R}}^{m\times d}$ is the weight matrix for input transformation, and $a_{c_*,j}^k$ is the corresponding k-th normalized attention coefficient.

$\textit{Dictionary embedding.}$ Dictionary embedding is constructed from a symptom dictionary $D_s$, which contains all symptoms in EMR data. For each symptom $s_i\in s_t^{(n)},$ its dictionary embedding $d_{s_i}$ is just its index value in $D_s$.

### Pre-training

The pre-training module creates a pre-training visit model based on the input embedding transformed from single-visit records of EMR. By pre-training, a large number of single visit data are effectively used to mine the richer internal features of EMR.

Before pre-training, a multi-layer Transformer architecture [50] is adopted to derive visit embedding from two ontology embedding and one dictionary embedding of each EMR data. For $P_t^{(n)}$, three types of visit embedding can be obtained as follows:

2 $$\begin{aligned} v_d^t= & {} Transformer\left( \left\{ [CLS]\right\} \cup \left\{ o_{d_i}\mid d_i\in d_t^{(n)}\right\} \right) \end{aligned}$$

3 $$\begin{aligned} v_m^t= & {} Transformer\left( \left\{ [CLS]\right\} \cup \left\{ o_{m_i}\mid m_i\in m_t^{(n)}\right\} \right) \end{aligned}$$

4 $$\begin{aligned} v_s^t= & {} Transformer\left( \left\{ [CLS]\right\} \cup \left\{ d_{s_i}\mid s_i\in s_t^{(n)}\right\} \right) \end{aligned}$$

where $v_d^t$ is diagnostic visit embedding, $v_m^t$ is drug visit embedding, $v_s^t$ is symptom visit embedding, and [CLS] is the first tag of each sequence whose final hidden state will be used as an aggregate sequence representation of the classification task for enabling BERT to better handle various downstream tasks. In order to obtain the consistent length of the input token, it is necessary to align the tokens obtained by padding.

This paper conducts the following two kinds of pre-training tasks to make visit embedding absorb enough information about medication recommendation.

$\textit{Mask EMR Field Task (Mask EF Task).}$ This task randomly masks some of the embedding to better represent information about the composition of EMR records. By changing word token masking of sentences [51] into field masking of EMR records, the following loss function is calculated:

5 $$\begin{aligned} \mathrm {L}_{s}\left( v_*,C_*^{(n)}\right) =-logP\left( C_*^{(n)}\mid v_*\right) =-\sum _{c_*\in c_*^{(n)}} logP(c_*\mid v_*)+\sum _{c_*\in (c_*\setminus c_*^{(n)})} logP(c_*\mid v_*) \end{aligned}$$

where $C_*^{(n)}=\left( d_t^{(n)} \cup m_t^{(n)} \cup s_t^{(n)}\right)$ is an union set of medical codes and symptoms of n-th patient, $c_* \in C_*^{(n)}$ denotes a medical code or symptom involved in the n-th patient and $c_*\in \left\{ c_*\setminus c_*^{(n)}\right\}$denotes the medical codes or symptoms not used for the n-th patient, $*\in \left\{ d,m,s\right\}$. We minimize the binary cross entropy loss $L_s$ to make the model have stronger self-prediction ability.

$\textit{Correlation Prediction Task (CorP Task).}$ This task is used to represent information about the correlation among diagnostic codes, drug codes and symptoms. In BERT, the next sentence prediction (NSP) task facilitates the prediction of sentence relations. G-Bert revised the NSP task as the multidirectional prediction task for predicting unknown disease or drug codes of the sequence [16]. This paper revises the NSP task [52] as the CorP Task. For mutual prediction of diagnostic codes, drug codes and symptoms, the following three loss functions are calculated:

6 $$\begin{aligned} \mathrm {L}_{dm}= & {} -logP\left( C_d^{(n)}\mid v_m\right) -logP\left( C_m^{(n)}\mid v_d\right) \end{aligned}$$

7 $$\begin{aligned} \mathrm {L}_{ds}= & {} -logP\left( C_d^{(n)}\mid v_s\right) -logP\left( C_s^{(n)}\mid v_d\right) \end{aligned}$$

8 $$\begin{aligned} \mathrm {L}_{ms}= & {} -logP\left( C_m^{(n)}\mid v_s\right) -logP\left( C_s^{(n)}\mid v_m\right) \end{aligned}$$

Finally, the pre-training optimization objective can simply be the combination of the aforementioned losses:

9 $$\begin{aligned} \mathrm {L}_{pr}=\mathrm {L}_{s}\left( v_d,C_d^{(n)}\right) +\mathrm {L}_{s}\left( v_m,C_m^{(n)}\right) +\mathrm {L}_{s}\left( v_s,C_s^{(n)}\right) +\mathrm {L}_{dm}+\mathrm {L}_{s}+\mathrm {L}_{ms} \end{aligned}$$

### Prediction

A MLP module with adversarial training is used to achieve the final prediction task. Based on the pre-training model, multi-visit EMR sequences can be transformed to three types of visit embedding sequences. Concatenating the average of previous diagnostic visit embedding, drug visit embedding, and symptom visit embedding before the t-th visit, as well as the diagnostic visit embedding and symptom visit embedding at the t-th visit, the MLP [53] can predict the recommended drug codes at the t-th visit as follows:

10 $$\begin{aligned} y_t=Sigmoid\left( W\left[ \left( \frac{1}{t-1} \sum _{\tau<t}v_d^{\tau }\right) \parallel \left( \frac{1}{t-1} \sum _{\tau<t}v_s^{\tau }\right) \parallel \left( \frac{1}{t-1} \sum _{\tau <t}v_m^{\tau }\right) \parallel v_d^{\tau } \parallel v_s^{\tau }\right] +b\right) \end{aligned}$$

where $W\in {\mathbb {R}}^{\mid C_m \mid \times 3l}$is a learnable transformation matrix.

Therefore, the loss function can be calculated as follows:

11 $$\begin{aligned} L_n=-\frac{1}{T-1} \sum _{t=2}^T\left( y_t^T log\hat{(y_t)}+\left( 1-y_t^T\right) log\left( 1-y_t^T\right) \right) \end{aligned}$$

where y is the predicted value sequence and ${\hat{y}}$ is the true value sequence. In this formula, t =2 means that the prediction starts from the second visit of the patient. The reason is that this paper focuses on longitudinal sequential medication recommendation which predicts the drugs currently suitable for the patient based on the patient’s historical and current diagnosis and symptom.

In order to avoid the over-fitting of model, this paper integrates the adversarial training FGM into the deep prediction model [54]. Adversarial training can not only improve the defense ability of the model against adversarial samples, but also improve the generalization ability of the original samples. For the prediction task, the disturbance $r_{adv-d}$ and $r_{adv-m}$ are added to the diagnostic ontology embedding and the drug ontology embedding respectively, in order to make the model wrong as much as possible and increase the robustness. Referring to [54], the disturbance can be calculated as follows:

12 $$\begin{aligned} \begin{aligned} r_{adv-d}=-\epsilon \frac{ \triangledown _x v_d^t}{\parallel \triangledown _x v_d^t \parallel _2} \\ r_{adv-m}=-\epsilon \frac{\triangledown _x v_m^t}{ \parallel \triangledown _x v_m^t \parallel _2} \end{aligned} \end{aligned}$$

where $\epsilon$ is a constant. $r_{adv-d}$ and $r_{adv-m}$ are normalized values with the gradient of $v_d^t$ and $v_m^t$. The drug sequence $y_t$ is predicted from the disturbed $v_d^{\tau '}$ and $v_m^{\tau '}$ which can be combined with the real drug sequence $\hat{y_t}$ to construct a loss function. In back propagation, the gradient of counter training is accumulated on the basis of the normal gradient. Then the original values of $v_d^{\tau }$ and $v_m^{\tau }$ are restored. Finally, the parameters are updated according to the gradient of accumulated adversarial training. The loss function after adversarial training is defined in the same way as Eq. (11) where $y_t$ is calculated from the disturbed diagnostic ontology embedding and drug ontology embedding on the basis of Eq. (13) as follows:

13 $$\begin{aligned} \begin{aligned} y_t=Sigmoid\left( W\left[ \left( \frac{1}{t-1} \sum _{\tau<t}\left( v_d^{\tau }+r_{adv-d}\right) \right) \parallel \left( \frac{1}{t-1} \sum _{\tau<t}v_s^{\tau }\right) \parallel \right. \right. \\\left. \left. \left( \frac{1}{t-1} \sum _{\tau <t}\left( v_m^{\tau }+r_{adv-m}\right) \right) \parallel \left( v_d^{\tau }+r_{adv-d}\right) \parallel v_s^{\tau }\right] +b\right) \end{aligned} \end{aligned}$$

## Experiment

### Baselines

We compared the proposed MR-KPA with the following baseline methods. All methods were developed under Pytorch and implemented on Nvidia Quadro P2000:

- Learn to Prescribe (LEAP) [34]: LEAP is an example based model that aims to prescribe effective and safe drug combinations for patients with recurrent diseases. It uses cyclic decoders to model labels and captures label instance maps using content-based attention in order to decompose treatment recommendations into a continuous decision-making process while automatically determining the appropriate drug quantity. The epoch of this model is set as 30.
- Logistic Regression (LR) [55]: This study adopts a logistic regression model with L1/L2 regularization as the baseline method. We represented sequential multiple medical codes by summing up multiple hot vectors per visit.
- Reverse Time Attention Model (RETAIN) [17]: RETAIN is a medication recommendation model based on a two-stage neuro attention that examines past influential visits and important clinical variables such as critical diagnoses within those visits. In this study, the epoch of the model is set to 30 which has the best performance by experiment. When the model predicts that the probability of a drug being recommended is greater than 30$\%$, the drug is recommended.

### Metrics

This paper uses the Jaccard Similarity Coefficient [56] and average F1 [57] to measure experimental results. They can be calculated as follows:

14 $$\begin{aligned} Jaccard=\frac{1}{\sum _k^N \sum _t^{T_k}1} \sum _k^N \sum _t^{T_k} \frac{\mid Y_t^{(k)} \cap {\hat{Y}}_t^{(k)}\mid }{\mid Y_t^{(k)} \cup {\hat{Y}}_t^{(k)}\mid } \end{aligned}$$

where ${\hat{Y}}_t^{(k)}$ is the predicted set and $Y_t^{(k)}$ is the ground truth set.

15 $$\begin{aligned} F1&=\frac{1}{\sum _k^N \sum _t^{T_k}1} \sum _k^N \sum _t^{T_k} \frac{2 \times P_t^{(k)} \times R_t^{(k)}}{P_t{(k)}+R_t^{(k)}} \end{aligned}$$

16 $$\begin{aligned} P_t^{(k)}&=\frac{\mid Y_t^{(k)} \cap {\hat{Y}}_t^{(k)}\mid }{\mid Y_t^{(k)} \mid }, R_t^{(k)}=\frac{\mid Y_t^{(k)} \cap {\hat{Y}}_t^{(k)}\mid }{\mid {\hat{Y}}_t^{(k)} \mid } \end{aligned}$$

where $P_t^{(k)}$ is the precision rate, $R_t^{(k)}$ is the recall rate, N is the number of patients in the test set and $T_k$ is the number of visit of the k-th patient. And we also use Precision Recall AUC (PR-AUC) to evaluate the performance of the algorithm.

### Implementations

We used all single-visit data for pre-training, and randomly divided multi-visit data into the training set, the verification set and the test set in a 4:1:1 ratio. We set the number of attention heads in the GAT model as 4, and the hidden layers in the pre-training model as 2 with 4 attention heads. In the prediction model, the learning rate was set as 5e-4. In this paper, the prediction was not made after the pre-training model was fully trained. Instead, the pre-training was carried out first, and then the prediction with the pre-trained model was made in alternate cycles, so as to artificially imitate the way of multi-task. Although the two models were not trained together, the two models influenced each other and improved each other in the cycle process, which effectively solved the problem of parameter forgetting of the pre-training model and effectively improved the model generalization ability.

### Results

Table 3 shows the performance results of different models. LEAP is obviously less effective than other baseline models and the proposed MR-KPA. As an instance-based medication recommendation model, LEAP does not take into account longitudinal EMR data. Therefore, this results prove that it is necessary to adopt the longitudinal sequential method, namely medication recommendation based on longitudinal EMR data in this study. LR is a shallow machine learning model and widely used in medication recommendation. RETAIN is a medication recommendation model based on the deep neural network. Compared with their results, the Jaccard score and PR-AUC score of LR are significantly higher than those of RETAIN. This indicates that, the deep learning models are no better than traditional shallow machine learning models based on the small-scale longitudinal EMR data. Therefore, it is also necessary to adopt the knowledge-enhanced pre-training visit model for realizing few-shot learning in this study. Finally, the proposed MR-KPA obtains the best results on all evaluating indicators. This shows that the proposed model can effectively improve the accuracy of medication recommendation based on small-scale longitudinal EMR data.

**Table 3:** Experimental results from MR-KPA and baselines

Methods	Jaccard	F1	PR-AUC
LEAP	0.0945	0.1188	0.1650
LR	0.1618	0.1722	0.4120
RETAIN	0.1254	0.2098	0.3069
MR-KPA	0.4482	0.5293	0.5889

## Discussion

Knowledge enhancement based on ontology embedding, the pre-training visit model and adversarial training are three core optimizations in this paper. This section will discuss their effectiveness by an ablation study. Seven MR-KPA variants are designed as follows:

- $MR-KPA_{K-,P-,A-}$: Compared with MR-KPA, this model deletes knowledge enhancement based on ontology embedding, the pre-training visit model and adversarial training, and only uses MLP to predict drug codes based on input embedding of diagnostic codes, drug codes and symptoms;
- $MR-KPA_{P-,A-}$: Compared to MR-KPA, this model deletes the pre-training visit model and adversarial training, and only keeps knowledge enhancement based on ontology embedding;
- $MR-KPA_{K-,A-}$: Compared to MR-KPA, this model deletes knowledge enhancement based on ontology embedding and adversarial training, and only keeps the pre-training visit model.
- $MR-KPA_{K-,P-}$: Compared to MR-KPA, this model deletes knowledge enhancement based on ontology embedding and the pre-training visit model, and only keeps adversarial training.
- $MR-KPA_{K-}$: Compared to MR-KPA, this model deletes knowledge enhancement based on ontology embedding, and keeps the pre-training visit model and adversarial training.
- $MR-KPA_{P-}$: Compared to MR-KPA, this model deletes the pre-training visit model, and keeps knowledge enhancement based on ontology embedding and adversarial training.
- $MR-KPA_{A-}$: Compared to MR-KPA, this model deletes adversarial training, and keeps knowledge enhancement based on ontology embedding and the pre-training visit model.

### All of three optimizations are effective and compatible

Table 4 gives the experimental results of the ablation study. Compare the baseline models, the result of $MR-KPA_{K-,P-,A-}$is similar to that of RETAIN. Its three evaluating indicators are significantly higher than those of LEAP and two evaluating indicators are lower than those of LR. This once again proves the necessity of adopting longitudinal sequential medication recommendation and the shortcomings of deep learning models in medication recommendation based on small-scale longitudinal EMR data.

**Table 4:** Experimental results of the ablation study

Methods	Jaccard	F1	PR-AUC
$MR-KPA_{K-,P-,A-}$	0.1553	0.2142	0.2792
$MR-KPA_{P-,A-}$	0.1720	0.2304	0.2860
$MR-KPA_{K-,A-}$	0.3266	0.4151	0.4899
$MR-KPA_{K-,P-}$	0.2184	0.2853	0.3348
$MR-KPA_{K-}$	0.4037	0.4893	0.5515
$MR-KPA_{P-}$	0.2275	0.2966	0.3791
$MR-KPA_{A-}$	0.3643	0.4570	0.5392
$MR-KPA$	0.4482	0.5293	0.5889

Compared with $MR-KPA_{K-,P-,A-}$, the result of $MR-KPA_{P-,A-}$,$MR-KPA_{K-,A-}$ and $MR-KPA_{K-,P-}$ achieve the better performance, which indicates that knowledge enhancement based on ontology embedding, the pre-training visit model and adversarial training, which are three core optimizations in this paper, are very effective. Furthermore, the results of $MR-KPA_{K-}$, $MR-KPA_{P-}$ and $MR-KPA_{A-}$ are also significantly improved than those of $MR-KPA_{K-,P-,A-}$. Finally, the proposed MR-KPA model achieved the best results. This indicates that these three optimizations are compatible with each other and their combination can greatly improve EMR-based medication recommendation.

### The pre-training visit model are the most effective optimization

**Fig. 4:**
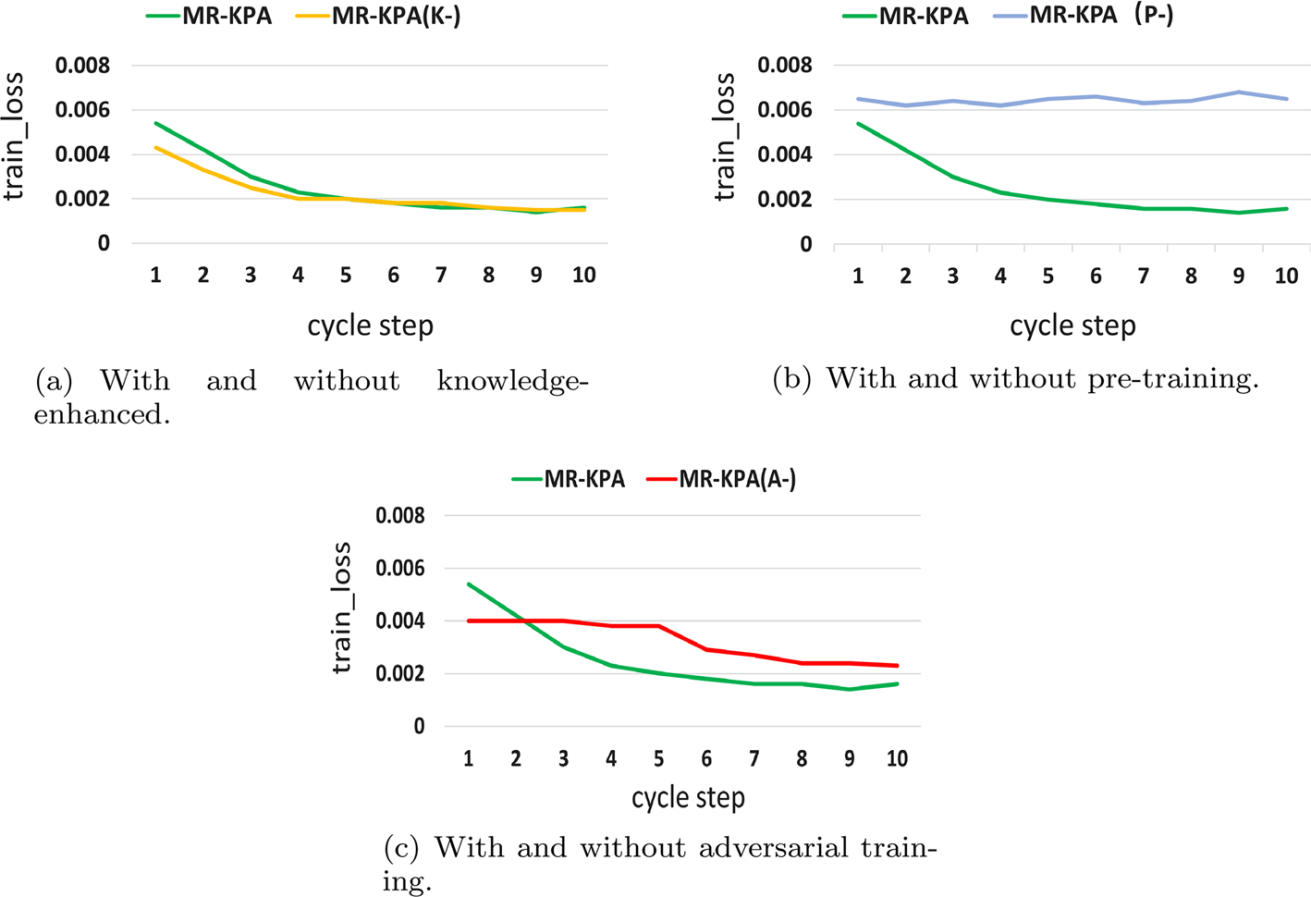
The change of training loss values in MR-KPA and three variants

Referring to [54], this section will further discuss the training effects of the three optimizations through the analysis of training loss curve. Figure 4 gives the learning curves of training loss of MR-KPA, $MR-KPA_{K-}$, $MR-KPA_{P-}$ and $MR-KPA_{A-}$. As shown in Fig. 4a, the training loss of $MR-KPA_{K-}$ drops a little faster than that of MR-KPA in the early stage, but it basically fits the training loss curve of MR-KPA in the later stage. This indicates knowledge enhancement based on ontology embedding affects the training speed in the early stage, but it has little impact on the recommendation results of the whole model. This is consistent with the results in Table 3. $MR-KPA_{K-}$ has the closest result to MR-KPA. This indicates that knowledge enhancement based on ontology embedding has the minimal improvement effect on the EMR-based medication recommendation task.

Figure 4b gives the comparison of training loss between MR-KPA and $MR-KPA_{P-}$. With the increase of iteration times, the loss of MR-KPA gradually decreased, but the loss change of $MR-KPA_{P-}$ is not obvious. This indicates that the pre-training visit model is the key to ensure the convergence of the model on relatively small-scale longitudinal EMR data. It has a significant effect on improving the edication recommendation based on small-scale longitudinal EMR data. This is also consistent with the results in Table 3. Among $MR-KPA_{K-}$,$MR-KPA_{P-}$ and $MR-KPA_{A-}$, $MR-KPA_{P}$ has the worst results.

Figure 4c gives the comparison of training loss between MR-KPA and $MR-KPA_{A-}$. With the increase of iteration times, the downward trend of loss of $MR-KPA_{A-}$is much slower than that of MR-KPA. The loss values MR-KPA are always below that of $MR-KPA_{A-}$ in the later. This indicates adversarial training has played a role in preventing the model from over-fitting on small-scale longitudinal EMR data. Therefore, it can effectively improve medication recommendation based on small-scale longitudinal EMR data, as shown in Table 3.

Comparing Fig. 4a–c, only the loss curve of $MR-KPA_{P-}$ decreases slowly, and even has an upward trend in the later period, indicating that the model does not converge. Therefore, $MR-KPA_{P-}$ has the worst result among $MR-KPA_{K-}$,$MR-KPA_{P-}$ and $MR-KPA_{A-}$, as shown in Table 3. That is to say, the pre-training visit model are the most effective optimization in this study.

### Limitations of this study

There are still some limitations in this study. Due to the addition of adversarial training, the computational complexity of the proposed MR-KPA inevitably increases, and the running time also increases. However, due to the small-scale training data, this limitation of recommendation model can be compensated partly. Another limitation of this study is that the temporal features of longitudinal data are not fully utilized. Therefore, an important future work is to effectively mine temporal features by various deep neural network, such as linear networks.

## Conclusion

In this paper, we propose a new EMR-based medication recommendation model called MR-KPA. By combining knowledge-enhanced pre-training with the deep adversarial network, MR-KPA improves both feature representation and the fine-tuning process to realize effectively medication recommendation based on small-scale EMR data. To our best knowledge, MR-KPA is real first that integrates current popular graph neural network, pre-training and adversarial training for EMR-based medication recommendation. The ablation experiments and comparative experiments prove that these three technologies are complementary and their integration makes the proposed MR-KPA model effectively realize medication recommendation on small-scale longitudinal EMR data. By reducing the dependence on high-quality labelled data, this study can greatly reduce the time and economic costs required for model construction, and help to promote the comprehensive application of EMRs based medication recommendation.

## Data Availability

The datasets generated and analysed during the current study are not publicly available due to privacy restriction from hospitals but are available from the corresponding author on reasonable request. The source codes are publicly available in the GitHub repository, https://github.com/MengzhenWangmz/MR-KPA.
